# Serological Profiling of Pneumococcal Proteins Reveals Unique Patterns of Acquisition, Maintenance, and Waning of Antibodies Throughout Life

**DOI:** 10.1093/infdis/jiae216

**Published:** 2024-04-29

**Authors:** Samantha W J He, Franziska Voß, Mioara A Nicolaie, Jolanda Brummelman, Martijn D B van de Garde, Elske Bijvank, Martien Poelen, Alienke J Wijmenga-Monsuur, Anne L Wyllie, Krzysztof Trzciński, Josine Van Beek, Nynke Y Rots, Gerco den Hartog, Sven Hammerschmidt, Cécile A C M van Els

**Affiliations:** Centre for Infectious Disease Control, National Institute for Public Health and the Environment, Bilthoven, The Netherlands; Department of Molecular Genetics and Infection Biology, Interfaculty Institute of Genetics and Functional Genomics, Center for Functional Genomics of Microbes, University of Greifswald, Greifswald, Germany; Centre for Infectious Disease Control, National Institute for Public Health and the Environment, Bilthoven, The Netherlands; Centre for Infectious Disease Control, National Institute for Public Health and the Environment, Bilthoven, The Netherlands; Centre for Infectious Disease Control, National Institute for Public Health and the Environment, Bilthoven, The Netherlands; Centre for Infectious Disease Control, National Institute for Public Health and the Environment, Bilthoven, The Netherlands; Centre for Infectious Disease Control, National Institute for Public Health and the Environment, Bilthoven, The Netherlands; Centre for Infectious Disease Control, National Institute for Public Health and the Environment, Bilthoven, The Netherlands; Department of Pediatric Immunology and Infectious Diseases, Wilhelmina Children's Hospital, University Medical Center Utrecht, Utrecht, Netherlands; Department of Pediatric Immunology and Infectious Diseases, Wilhelmina Children's Hospital, University Medical Center Utrecht, Utrecht, Netherlands; Centre for Infectious Disease Control, National Institute for Public Health and the Environment, Bilthoven, The Netherlands; Centre for Infectious Disease Control, National Institute for Public Health and the Environment, Bilthoven, The Netherlands; Centre for Infectious Disease Control, National Institute for Public Health and the Environment, Bilthoven, The Netherlands; Laboratory of Medical Immunology, Radboudumc, Nijmegen, The Netherlands; Department of Molecular Genetics and Infection Biology, Interfaculty Institute of Genetics and Functional Genomics, Center for Functional Genomics of Microbes, University of Greifswald, Greifswald, Germany; Centre for Infectious Disease Control, National Institute for Public Health and the Environment, Bilthoven, The Netherlands; Infectious Diseases and Immunology, Department of Biomolecular Health Sciences, Faculty of Veterinary Medicine, Utrecht University, Utrecht, The Netherlands

**Keywords:** *Streptococcus pneumoniae*, aging, pneumococcal proteins, pneumococcal carriage, antibodies

## Abstract

*Streptococcus pneumoniae* is a leading cause of morbidity and mortality in children and older adults. However, knowledge on the development of pneumococcal protein-specific antibody responses throughout life is limited. To investigate this, we measured serum immunoglobulin G (IgG) levels to 55 pneumococcal proteins in 11-month-old infants (n = 73), 24-month-old children (n = 101), parents (n = 99), adults without children <6 years of age (n = 99), and older adults aged >60 years (n = 100). Our findings revealed low IgG levels in infancy, with distinct development patterns peaking in adults. A decrease in levels was observed for 27 antigens towards older age. Adults and older adults had increased IgG levels during pneumococcal carriage and at increased exposure risk to *S. pneumoniae*. Carriage was a stronger predictor than exposure or age for antibody responses. These findings highlight the dynamic nature of naturally acquired humoral immunity to pneumococcal proteins throughout life, offering insights for age-targeted interventions.

**Clinical Trials Registration:**

Participants were selected from three clinical studies (NTR3462, NTR5405 and NTR3386) conducted in the Netherlands by the National Institute for Public Health and the Environment (RIVM).

## Teaser

Pneumococcal protein-specific antibody immunity develops via distinct patterns with selective waning at older age. Pneumococcal carriage is a stronger predictor of antibody levels than age and frequent exposure. Increased pneumococcal exposure risk in adults is associated with enhanced antibody levels.

*Streptococcus pneumoniae* (pneumococcus) is one of the leading causes of morbidity and mortality worldwide. The onset of pneumococcal disease is preceded by colonization of the upper airways. Pneumococcal carriage is predominantly observed in children ≤5 years old, who are therefore considered the primary reservoir for this pathobiont. Concurrently, high incidence of invasive pneumococcal disease (IPD) is reported in this age group with subsequent decrease with age, and increasing again in individuals over 65 years old [1, 2].

Antibodies play a pivotal role in protection against *S. pneumoniae* by targeting capsular polysaccharide serotypes (CPS) as well as intra- and extracellular proteins [3–7]. These antibodies exert various protective mechanisms, including neutralizing virulence factors and enhancing opsonophagocytosis, ultimately clearing the bacterium or preventing colonization [8–10]. Hence, the introduction of CPS-based pneumococcal conjugate vaccines (PCVs) effectively reduced IPD caused by vaccine serotypes [11]. However, their impact is hindered by serotype replacement and pneumococcal capsule switching [11–13]. This prompted growing interest in pneumococcal proteins as antigens in next-generation vaccines, the development of which has proven to be challenging due to the lack of correlates of protection and failure so far to prevent colonization or disease in clinical trials [9, 14–16].

A significant factor contributing to this is our limited grasp of how humoral immunity develops in response to individual pneumococcal proteins. The pneumococcal proteome, comprising diverse protein classes with distinct, occasionally overlapping, functionalities and intrinsic immunogenic properties, presents a complex landscape for the immune system to target. Early-life pneumococcal colonization initiates primary antibody responses to multiple proteins in parallel, while subsequent carriage episodes with age boost quantity and avidity of these antibodies [17]. Whilst previous studies endeavored to address this critical knowledge gap, their focus was often restricted to proteins considered as potential vaccine candidates or specific age groups [7, 18–24].

Here, we aimed to provide an in-depth, comparative and comprehensive insight into the development, maintenance, and potential waning of antibody responses to a panel of 55 antigens, encompassing several protein classes and selected based on the critical functions in pneumococcal fitness and/or virulence. The selection included several vaccine candidates with demonstrated protective capacity in preclinical animal models and immunogenicity in clinical studies [25–29]. Antibody levels were determined in 11-month-old infants, 24-month-old children, parents, aged-matched adults without children <6 years old, and older adults >60 years old. We also investigated the impact of pneumococcal colonization and contact with children <6 years old on the protein-specific antibody levels.

## METHODS

### Ethics Statement

Participants were selected from 3 prospective cross-sectional studies (NTR3462, registered 3 September 2012) [30], NTR5405, registered 18 August 2015 [11], and NTR3386, registered 26 April 2011 [31]) conducted in the Netherlands by the National Institute for Public Health and the Environment (RIVM) and registered at OMON (https://onderzoekmetmensen.nl). All studies were approved by the independent medical ethics committee for reviewing research involving human subjects. Written informed consent for sampling of venous blood, nasopharyngeal swab, and structured interview (Supplementary Table 1) during home visit was obtained from all participants or their parents. All procedures were performed according to Good Clinical Practice and in accordance with the Declaration of Helsinki of the World Medical Association.

### Study Design

Eleven-month-old infants, 24-month-old children, parents of 24-month-old children, and age-matched adults without children <6 years old were selected from NTR3462 (1 October 2012 until 5 March 2013) [30] with additional serum samples from 24-month-old children from NTR5405 (1 September 2015 until 26 February 2016) [11]. Exclusion criteria were fever, craniofacial or chromosomal abnormalities, known or suspected immunodeficiencies, coagulation disorders, or use of anticoagulant medication. All 11-month-old children were immunized according to the national immunization program at the time with PCV10 (Synflorix) at 2, 3, and 4 months of age, whilst 24-month olds were vaccinated with PCV7 (Prevenar) or PCV10 at 2, 3, 4, and 11 months of age. Serum samples from community-dwelling older adults >60 years of age without respiratory symptoms were collected in NTR3386 [32] performed in the same period by the same study team as NTR3462. Adult participants did not receive pneumococcal vaccination, except for 1 adult and 15 older adults with unknown vaccination status. Serum samples were selected from these studies to achieve equal distribution of sex and pneumococcal carriage across groups, wherever sample availability allowed. Pneumococcal carriage was established based on conventional culture and culture-enriched quantitative polymerase chain reaction (qPCR) detection using identical procedures by the same laboratory for all studies, NTR3462 and NTR5405 [11, 30, 31, 33], as well as NTR3386 [34]. Testing positive with either method was considered as positive detection of *S. pneumoniae*.

### Heterologous Expression and Purification of Recombinant Pneumococcal Antigens

Fifty-five pneumococcal proteins, known or anticipated to be immunogenic or essential for protection, were recombinantly expressed as N-terminally His_6_-tagged full-length proteins without signal peptide and hydrophobic cell wall-anchoring regions. Purification was performed under native conditions followed by His-tag–mediated immobilization to beads for optimal structural integrity in the bead-based xMAP multiplex platform (Luminex) (Supplementary Table 2). The panel encompassed protein classes including (nonlipidated) lipoproteins, choline-binding proteins, sortase-anchored proteins, cytoplasmic proteins, membrane-associated protein, and others. Amongst them are several preclinical and clinical vaccine candidates (references in Supplementary Table 2). Target gene cloning, heterologous protein expression in *Escherichia coli,* and affinity chromatography purification procedures have been recently published or performed accordingly for newly produced proteins (No. 20–23, 44, 54, 55) [25].

### Detection of IgG Levels

Immunoglobulin G (IgG) levels in sera were measured with fluorescent bead-based multiplex immunoassay [25, 35]. Identical batches of proteins and beads were utilized for all measurement to prevent batch-induced differences. Coupling controls were performed to confirm the yield of the coupling of antigens on beads using mouse anti-His_5_ antibody (34660; Qiagen) and secondary R-phycoerythrin (RPE)-conjugated anti-mouse antibody (115-116-146; Dianova). Seven-serial dilutions of serum samples (from 1:50 to 1:204 800) were incubated with the bead mixture overnight alongside a bead mixture with phosphate-buffered saline (PBS) as blank. After washing (3 times with PBS plus 0.05% Tween 20 [P7949; Sigma]), RPE-conjugated anti-human IgG antibody (109-116-098; Dianova) was added for 90 minutes. Then, samples were washed (3 times with PBS plus 0.05% Tween 20 [P7949; Sigma]), resuspended in xMAP Sheath Fluid (4050015; Thermo), and measured on a Flexmap 3D (Luminex).

IgG levels were calculated using the xMAPr pipeline (https://github.com/stemicha/xMAPr_app) [35]. Measured samples were filtered for sufficient bead counts, blank values subtracted, and normalized with coupling controls for comparisons between antigens [35]. A saturation curve model was fitted through all dilution points to estimate the half-maximal mean fluorescence intensity (MFI) per protein. Antibody levels were calculated as the product of x-fold dilution of the half-maximal MFI and the half-maximal itself.

### Statistical Analyses

Antibody levels were log_10_-transformed and analyzed in R (version 4.1.1). Missing data were removed list-wise. Participants were stratified by sex, carriage, and pneumococcal exposure to control for confounding factors to explore the impact of age (Supplementary Table 1). Exposure risks for children aged 11 and 24 month was defined as attending day care (>1 day a week) and/or living with siblings < 6 years old. Parents were categorized as experiencing increased exposure risk to pneumococcus, while age-matched adults without children <6 years old were considered to have lower exposure risk. Older adults with monthly or rare contact with children < 6 years old were classified as minimal exposure risk group, whereas daily or weekly contact with children <6 years old were considered increased exposure risks. Age-related differences in IgG levels were analyzed via Wilcoxon rank sum test with Benjamini-Hochberg correction for multiple testing and a false discovery rate of 5%. The association between sex, carriage, pneumococcal exposure, and antibody levels within each age group was assessed by Mood's median nonparametric test, followed by post hoc analysis.

Random forest analysis was used to identify factors that could predict the IgG levels (randomForest package, version 4.7.1). One thousand trees were used and the value for the number of variables sampled for each split was optimized to yield the smallest out-of-bag prediction error.

## RESULTS

### Study Population

Serum samples were selected from participants in 3 cross-sectional studies to investigate age-related patterns in pneumococcal protein-specific antibodies (Table 1). Whilst balanced distribution for sex was more or less achieved for each group, pneumococcal carriers remained overrepresented among the children (69.9% and 68.3% in 11-month and 24-month olds, respectively) and underrepresented in adults without young children (21.2%).

**Table 1.** Study Population

Characteristic	Age Group				
	11 mo	24 mo	Parents	Adults	Older Adults
No. of Individuals	73	101	99	99	100
Age, d or y, mean (SD)	331.3 d (22.0)	742.2 d (19.5)	36.2 y (4.4)	39.5 y (8.7)	71.1 y (8.0)
Female	39 (53.4)	48 (47.5)	60 (60.6)	49 (49.5)	50 (50.0)
Pneumococcal carriers	51 (69.9)	69 (68.3)	49 (49.5)	21 (21.2)	50 (50.0)
Pneumococcal carriers in the source population [31, 33]	191 (65.0)	185 (63.0)	29 (10.0)	5 (2.0)	Not published
Frequent pneumococcal exposure	57 (78.1)	77 (76.2)	99 (100.0)	0 (0.0)	39 (39.0)

Data are No. (%) except where indicated.

### Serum IgG Levels Vary Between Pneumococcal Protein

**Figure 1.**
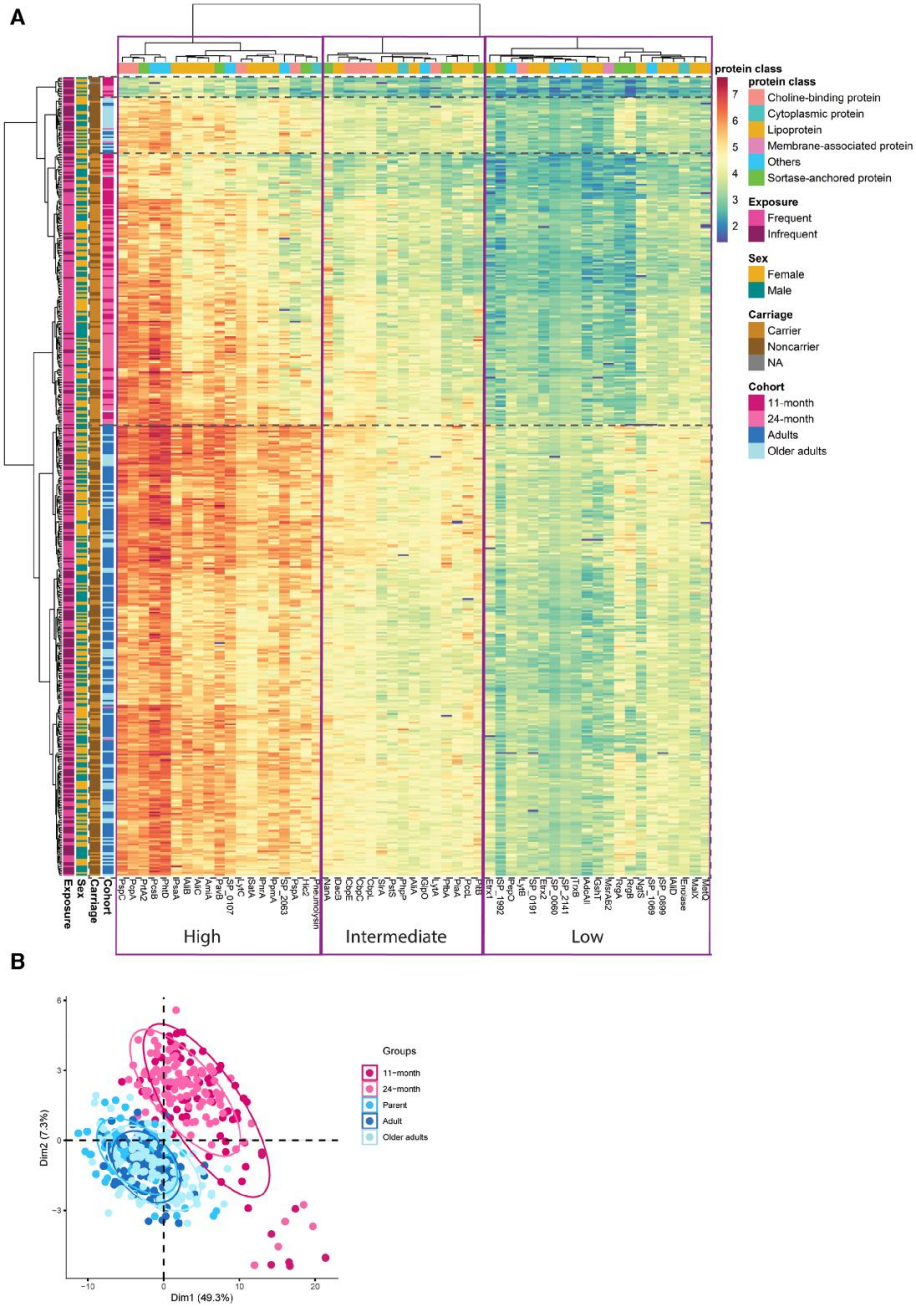
Serological profiling of antibody responses to pneumococcal proteins in age groups. *A*, Heatmap of normalized and log_10_ transformed IgG levels against a 55-multiplex pneumococcal protein panel in 476 serum samples. Each row represents 1 serum sample, while each column represents an individual protein in the panel. The colors in the heatmap indicate low to high IgG levels. The age group, pneumococcal carriage status, sex, and pneumococcal exposure risk of each sample are depicted by the colors on the left of the heatmap, while protein class of each protein are indicated on top of the heatmap. Hierarchical clustering was performed using Ward's method. *B*, Principal component analysis of the IgG levels against the pneumococcal proteins in age groups. Each dot represents 1 serum sample and the colors represent the age groups. The percentage of variance explained by each principal component is indicated on the axes.

To gain insight into the development of protein-specific antibody responses throughout life, we measured serum IgG levels in different age groups against 55 pneumococcal proteins. Most proteins were immunogenic as reflected by the presence of IgG detected across age groups (Figure 1*A*). Heatmap analysis revealed highest IgG levels for 19 proteins (34.5%) across all age groups, encompassing all clinical and several preclinical vaccine candidates. Notably, within the group exhibiting high IgG levels are 8 pneumococcal proteins, AliB, AliC, AmiA, Hic2, PavB, SP_0107, and SP_2063, whose protective potential has yet to be fully explored in preclinical settings as vaccine candidates. Intermediate IgG levels were noted for 15 proteins (27.3%), while relatively lower IgG levels were observed for the remaining 21 proteins (38.2%), including RrgA and RrgB (subunits of pilus type-1). Clustering of the heatmap (Figure 1*A*) further indicated 4 distinct responder profiles (dotted lines in Figure 1*A*), mainly separating adults and children and 2 smaller groups of noncolonized children and older adults. The clear separation between the adult groups and the children was further corroborated by principal component analysis (PCA) (Figure 1*B*). Manual sorting of proteins into their respective classes demonstrated that there was no discernible immunodominance associated with specific protein class. This was evident as all protein classes (with >1 protein) contained targets with high and low IgG levels (Supplementary Figure 1).

### Four Distinct Antibody Development Patterns From Early Childhood to Adulthood

**Figure 2.**
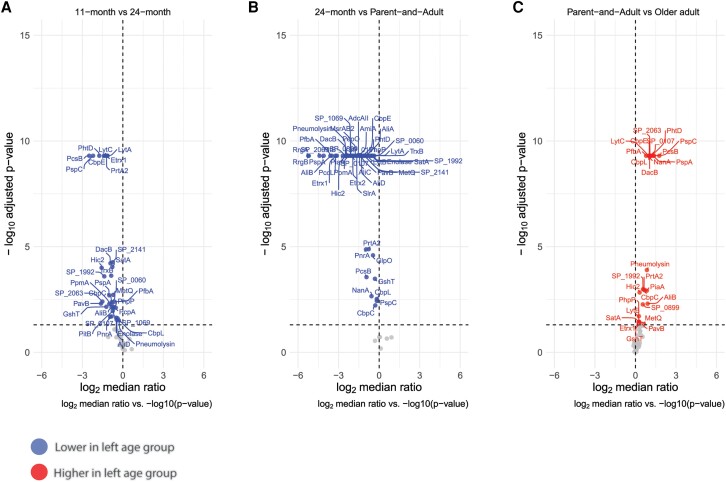
Comparative analysis of pneumococcal protein-specific IgG levels between consecutive age groups. Volcano plots depict log_2_-fold increments in IgG responses between 2 consecutive age groups for the protein panel after controlling for confounding factors such as sex, ongoing pneumococcal carriage episodes, and increased exposure risk. Y-axis depicts the log_10_-transformed *P* value (cutoff at .05 on the original scale and at log_10_ [.05] on the transformed scale), whilst the x-axis depicts the log_2_-fold difference in IgG levels against individual proteins between the indicated age groups: (*A*) 11-month versus 24-month-old children; (*B*) 24-month children versus parent-and-adult combined; and (*C*) parent-and-adult combined versus older adults (cutoff at −1 for proteins with at least 2-fold lower IgG levels in left age group, cut-off at +1 for proteins with at least 2-fold higher IgG levels in left age group, as color indicated). Colors of the dots indicate the log_2_-fold difference between the indicated age groups. Significance was based on the Wilcoxon rank sum test with Benjamin-Hochberg correction for multiple testing.

Evaluation of IgG levels’ development between consecutive age groups showed significant increases for 34 out of 55 pneumococcal proteins in 24-month-old compared to 11-month-old children (Figure 2*A*), despite overlap in PCA. Parents and age-matched adults without children < 6 years of age combined (parent-and-adult) exhibited higher IgG levels for 53 and 49 proteins compared to 11-month-old and 24-month-old children, respectively (Figure 2*B* and Supplementary Figure 2*A*).

**Figure 3.**
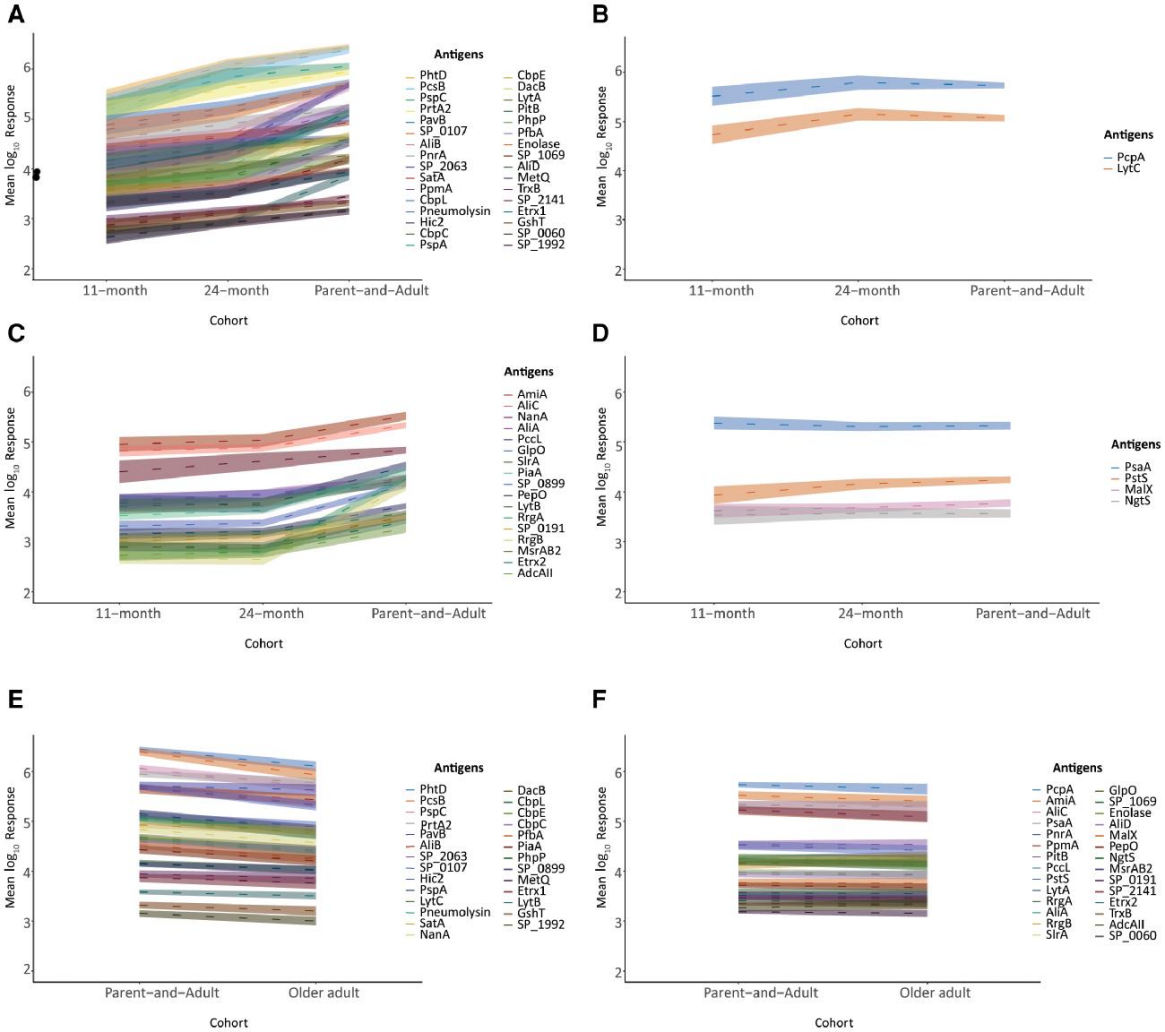
Developmental patterns and waning of pneumococcal-specific IgG levels with age. Line graph depicting changes in mean log_10_ pneumococcal protein-specific IgG levels with age based on (in)significant differences calculated for Volcano plots (Figure 2*B*): (*A*–C) changes from 11-month to parent-and-adult group; (*D*) four antigens that remained unchanged throughout life (no significant changes detected); (*E*) significant decrease in IgG levels to specific antigens at older age; and (*F*) antigens that remained constant between parent-and-adult group and older adults. Lines between groups serve as visualization of patterns, but do not indicate longitudinal relationship.

Four development patterns emerged based on statistically significant differences between 11-month, 24-month, and parent-and-adult groups (Figure 3*A*–*D* and Supplementary Figure 3). Gradual increase in IgG levels from the first years of life to adulthood was observed for 32 proteins (Figure 3*A*), including the majority of highly immunogenic proteins identified in the heatmap (Figure 1*A*). In contrast, IgG levels to preclinical candidates, PcpA and LytC, already reached adult-like levels at 24 months of age after an initial rise in the first 2 years of life (Figure 3*B*).

For the remaining 17 proteins, increases in IgG levels were only observed after 24 months of age into adulthood (Figure 3*C* and Supplementary Figure 3). Amongst them are highly immunogenic AmiA and AliC, together with preclinical vaccine candidates, AliA, PiaA, NanA, LytB, and GlpO. Notably, IgG levels to lipoproteins NgtS, PstS, PsaA (clinical vaccine candidate), and MalX (preclinical vaccine antigen) did not change significantly throughout life (Figure 3*D*).

### Decreased IgG Levels to Specific Pneumococcal Antigens at Older Age

To assess whether pneumococcal protein-specific antibody responses are maintained at older age, we compared IgG levels for each antigen between the parent-and-adult group and older adults. Significantly decreased IgG levels were observed at older age compared to the parent-and-adult group for 27 proteins, including the majority of highly immunogenic proteins (Figure 2*C* and Figure 3*E*). No significant changes were detected for the other 28 proteins (Figure 3*F*), including highly immunogenic PcpA, PsaA, AmiA, AliC, PnrA, and PpmA.

Most of the 27 proteins with decreased IgG levels at older age (Figure 3*E*) displayed the gradual developmental pattern from early childhood to adulthood (Figure 3*A*), except for 5 proteins. Antibodies targeting these proteins either reached adult-like IgG levels at 24 months of age (ie, LytC; Figure 3*B*), or increased after 24 months of age (ie, SP_0899, PiaA, LytB, and NanA; Figure 3*C*).

### Pneumococcal Carriage and Increased Exposure Risk Associate With Increased IgG Levels in Adults

Next, we investigated if sex, carriage status, and increased exposure risk were associated with IgG levels in the adult groups. Participants were stratified based on these 3 risk factors, thereby also separating parents (increased exposure risk) from age-matched adults without children <6 years old (lower exposure risk) (Supplementary Table 1). Median values were compared between strata to assess associations between risk factors and changes in IgG levels.

**Figure 4.**
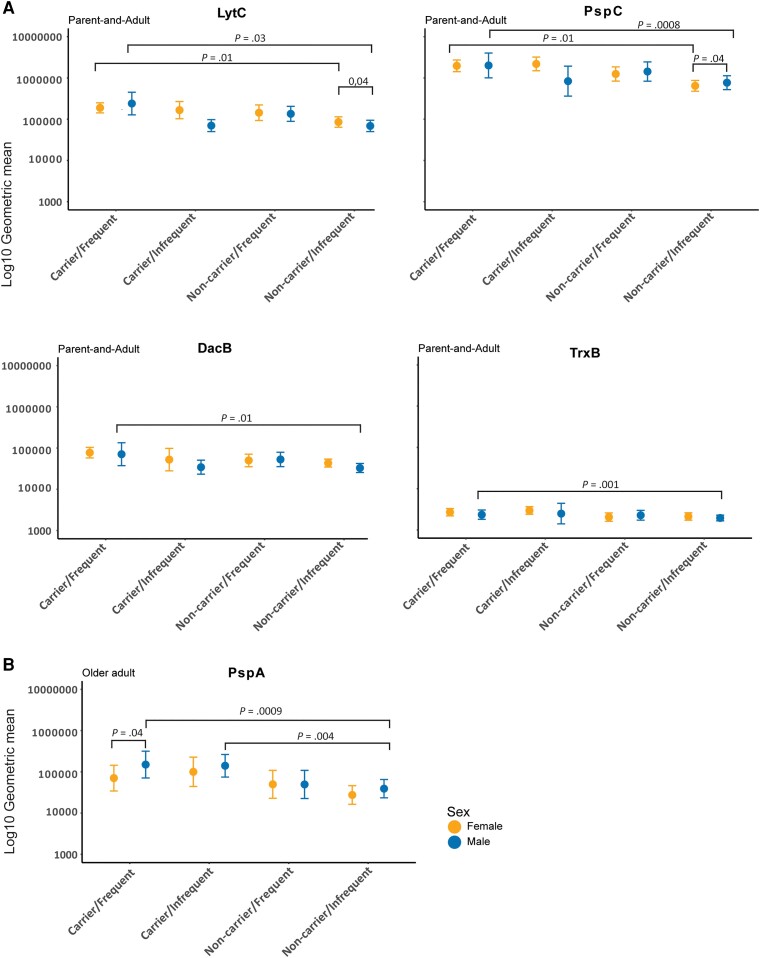
Impact of sex, pneumococcal carriage, and exposure on IgG levels within age groups. Parent-and-adult group and older adults were stratified by 3 risk factors, including sex, pneumococcal carriage status, and exposure frequency. Graph depicts the geometric mean of antibody levels on log_10_ scale to (*A*) LytC, PspC, DacB, and TrxB in parent-and-adult group; and (*B*) PspA in older adults. Significance was calculated by Mood's median and post hoc analysis. Error bars represent the confidence intervals of geometric mean. Partial significance is left out of graphs for visibility (see Supplementary Table 3 for full results).

Significant associations were found for LytC, PspC, DacB, and TrxB in adult groups (Figure 4 and Supplementary Table 3). The combination of pneumococcal carriage and increased exposure risk enhanced IgG levels for LytC and PspC in both sexes, but only in men for DacB and TrxB, compared to their respective counterparts that did not carry and had lower risk of pneumococcal exposure. In the absence of colonization and low exposure risk, women had higher antibody levels for LytC compared to men, while men had higher levels for PspC compared to women.

Older men carrying pneumococcus and experiencing lower exposure risk had higher IgG levels to PspA compared to older men not carrying pneumococcus at the moment of sampling (Figure 4). This elevated anti-PspA IgG levels in these older male carriers with increased risk of exposure were significantly higher than those in older adult women with similar risk factors. Analysis of IgG levels to individual proteins in children did not reveal differences in IgG levels to individual proteins between participants of different sexes, carriage status, or exposure risk (Supplementary Table 4).

### Pneumococcal Carriage Is an Important Predictor of Pneumococcal Protein-Specific Antibody Responses

**Figure 5.**
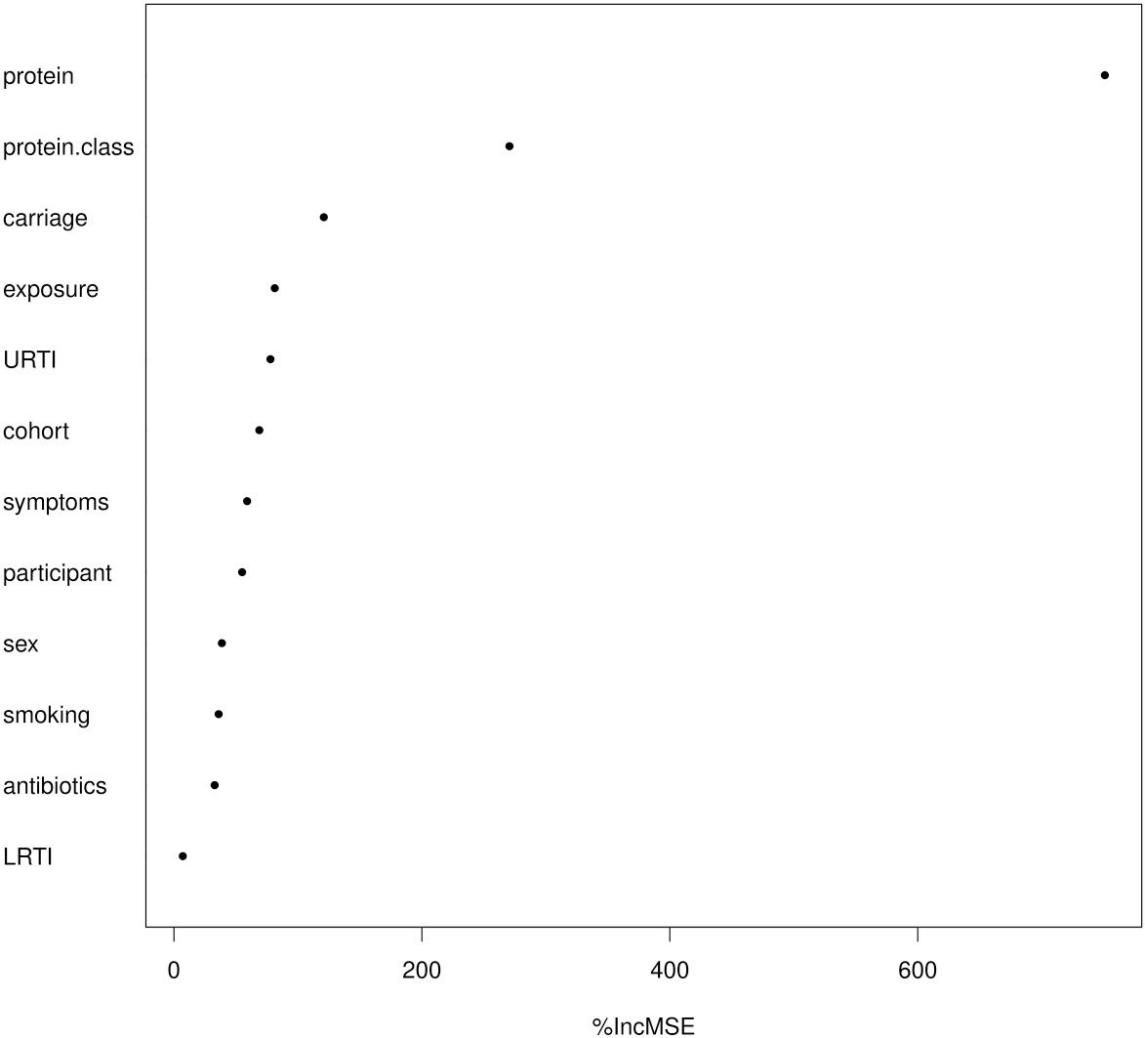
Random forest prediction of the impact of variables on pneumococcal protein-specific IgG levels. The figure shows the importance of variables in predicting pneumococcal protein-specific IgG levels, ranked by the percentage increase in mean square error (%IncMSE). The list of predictors includes protein, protein class, pneumococcal carriage, pneumococcal exposure frequency, cohort, infection in the upper (URTI) and lower respiratory tract (LRTI), ongoing infection (symptoms), smoking, sex, and antibiotic usage in the past 12 months (antibiotics).

We applied random forest analysis on pooled IgG levels from all age groups to assess the impact of the protein, protein class, age (cohort), pneumococcal carriage, risk of pneumococcal exposure, ongoing infections at the moment of blood sampling (symptoms), antibiotic use, and upper (URTI) and lower respiratory tract infection (LRTI) in the past 12 months (Figure 5 and Supplementary Table 1). Together, these variables accounted for 74% of the total variation in IgG levels with a mean absolute error of 0.96 (Supplementary Figure 4). Permutation importance ranked the protein as the most important variable in predicting IgG levels in our model, followed by protein class. Ongoing pneumococcal carriage ranked as the third important variable in predicting IgG levels, surpassing pneumococcal exposure risk, URTI, and age (cohort). This was followed by symptoms and other participant-related factors that were not measured in our study (participant). Sex, smoking, and antibiotic use had minor impact on predicting protein-specific IgG levels, while no evident for LRTI in prediction is found. Overall, our findings highlight the importance of ongoing pneumococcal carriage as main factor in predicting levels of pneumococcal protein-specific antibodies, above age and pneumococcal exposure risk.

## DISCUSSION

Insight into the development and persistence of pneumococcal protein-specific antibody levels with age is limited. We addressed this by conducting a comprehensive serological profile analysis of a wide range of pneumococcal proteins in age groups ranging from infants to older adults. We identified distinct patterns of development and waning of individual pneumococcal protein-specific antibodies throughout life, indicating a highly dynamic immune status that alters in strength and immunodominance with time. Ongoing carriage episodes and increased pneumococcal exposure risk were significantly associated with increased IgG levels in adults and older adults. Pneumococcal carriage at the time of sampling emerged as the most important clinical predictor for protein-specific antibody levels.

Humoral immunity to pneumococcus is expected to gradually develop by subsequent exposures with age. Indeed, recent data from our laboratory indicated an increase of levels and avidity of IgG and IgA antibodies to the full pneumococcal proteome between child- and adulthood [17]. Our current study corroborates the observed general quantitative trends in IgG, but also further reveals that development patterns may vary on an individual pneumococcal protein level. For nearly all tested pneumococcal proteins, IgG levels were the lowest in the first years of life and developed in 4 different patterns towards adulthood. Antibody levels to the majority of proteins (32 of 55), including multiple preclinical and clinical vaccine candidates, showed step-wise increase until adulthood, consistent with previous studies [6, 18, 19, 36, 37]. IgG levels to 17 other antigens remained relatively low up to 24 months old and eventually increased into adulthood, including RrgA, RrgB, and AliC (oligopeptide-binding protein of nonencapsulated pneumococci). Whilst these antigens are associated with enhanced virulence [38, 39], their presence in pneumococcal isolates are highly variable [40]. Multiple encounters may be needed to develop antibody responses to these proteins, possibly explaining the delayed developmental pattern. This was not applicable to the remaining 14 proteins with this development pattern as they are part of the pneumococcal core genome (Pangenome, Pneumowiki; https://pneumowiki.med.uni-greifswald.de/) and hence are expected to be present irrespective of isolates. As the random forest analysis showed protein as the strongest predictor of antibody responses, we propose that inherent immunogenic characteristics of the antigen itself, such as expression levels during colonization, accessibility, epitope count, or immune evasion properties, contribute to the observed differences in development patterns. Collectively, the lower responsiveness to a wide array of proteins in high-risk children is consistent with the importance of antibodies in protection against carriage and/or IPD as shown in animal models (references in Supplementary Table 2). However, conflicting results exist regarding the relationship between decreased carriage/pneumococcal disease and pneumococcal protein-specific antibody levels in young children [6, 41, 42]. This may result from studies focusing on too few rather than multiple proteins. Our study highlights this with lower levels across the protein panel, except for 4 proteins.

Antibodies to the remaining 6 proteins, PstS, NgtS, lytC, and preclinical vaccine candidates PcpA, PsaA, and MalX, reached steady levels in the first 2 years and were sustained until older age. High levels of PsaA-specific IgG antibodies from 11 months old onwards have been reported before in western and nonwestern countries [6, 36, 37, 43]. This suggests that initial encounters early in life with pneumococcus and nonpneumococcal streptococci expressing these antigens are sufficient to induce sustained antibody responses to certain proteins [44]. As further natural boosting of antibody levels to these proteins seemed absent, it remains to be seen whether vaccines based on these antigens can go beyond these naturally acquired levels.

We observed decreased antibody levels to half of the antigens in our panel at older age, including the majority of clinical and preclinical vaccine candidates. Similar decrease with older age was observed previously for PspA, PspC, PhtD, LytC, and NanA [19, 20]. As older adults face elevated risk of IPD, this indicates the potential impact of antibody waning in contributing to the heightened susceptibility. However, the maintained IgG levels to the other half of the panel and overall higher IgG levels compared to children also suggest that other immune mechanisms impacted by aging likely play a role in the increased susceptibility at older age as well, such as decline in innate responses [45], antibody functionality [46], waning of polysaccharide-specific antibody levels [19], or CD4^+^ T-cell responses [47].

Notably, Laine [18] reported no decline in PspA-specific antibodies in older individuals from Kenya, a country shown to have high carriage rates prior to the introduction of PCV10 in 2011 [48]. Interestingly, we observed increased IgG levels to DacB, TrxB, LytC, and PspC in adults and to PspA in older adults in the presence of pneumococcal carriage and exposure in our study, consistent with experimental colonization studies in corresponding age groups [21, 49]. Together, the observed association between higher IgG levels, carriage, and increased exposure risks suggests an important role for pneumococcal colonization in maintaining antibody responses at older age. This is further supported by pneumococcal carriage as the primary predictor of protein-specific antibody levels, surpassing age, and reinforcing pneumococcal colonization as an immunizing event.

Our study has several limitations. First, antibody levels reported in this study are relative due to absence of a reference. Second, antibody levels for antigens with high variation in the pneumococcal population (eg, PspA and PspC) may be underestimated. Third, although our study revealed immunogenic overlap with a diverse range of antigens consistent with previous research [22–24, 50], there are additional antigenic targets identified in those studies that were not included in our panel. These additional targets may also play a role in contributing to protective immunity. Finally, the impact of pneumococcal carriage or exposure risk on antibody responses might not be completely captured due to limited information on onset, frequency, density, and duration of carriage or incomplete identification of risk factors for exposure.

Overall, our study reveals distinct development patterns in young children and selective declines to pneumococcal proteins at older age. The association of relatively lower antibody levels and higher IPD incidences in infants and older adults underscores the importance of humoral immunity in protection against *S. pneumoniae*. The unique antibody development and decline patterns across various proteins also accentuate the critical significance of intrinsic protein immunogenicity and persistent humoral responses, which should be taken into account in the selection of protein-based vaccine candidates. Finally, our data suggest that continued exposure later in life may maintain antibody responses, and consequently contribute to protection, even amidst heightened risks of carriage and IPD. Together, we revealed how natural immunity to pneumococci develops and identified determinants for protein-based humoral immunity that provide insight for strategies to protect vulnerable groups of the population.

## Supplementary Data

Supplementary materials are available at *The Journal of Infectious Diseases* online (http://jid.oxfordjournals.org/). Supplementary materials consist of data provided by the author that are published to benefit the reader. The posted materials are not copyedited. The contents of all supplementary data are the sole responsibility of the authors. Questions or messages regarding errors should be addressed to the author.

## Supplementary Material

jiae216_Supplementary_Data

## Notes

***Author contributions.*** C. E., S. Ha., S. He., and F. V. designed the research. N. R., J. v. B., A. W. M., A. W., K. T., and E. B. were responsible for the clinical trials and pneumococcal carriage detection. S. He., M. P., and F. V. performed experiments. S. He., F. V., M. A. N., J. B., G. H., C. E., and S. Ha. analyzed and interpreted data. S. He., G. H., C. E., F. V., and S. Ha. wrote the manuscript. Critical revision of manuscript was provided by J. v. B., N. R., and M. G.

***Acknowledgment.*** We express our gratitude to the clinical teams and participants of the studies.

***Financial support*.** This work was supported by the Bundesministerium für Bildung und Forschung (BMBF/DLR) project Pneumofluidics (grant number FKZ 01DP19007 to S. Ha.); and BMBF Zwanzig20-InfectControl 2020 project VacoME (grant number FKZ 03ZZ0816A to S. Ha.).
