# Supplementary material for: Serological Profiling of Pneumococcal Proteins Reveals Unique Patterns of Acquisition, Maintenance, and Waning of Antibodies Throughout Life
Source: J Infect Dis. 2024 Apr 29;230(6):e1299–310. doi: 10.1093/infdis/jiae216 (PMC11646596; doi:10.1093/infdis/jiae216)
Supplement: jiae216_Supplementary_Data [file jiae216_supplementary_data.zip › Supplementary Figures.docx]

**Serological profiling of pneumococcal proteins reveals unique patterns of acquisition, maintenance and waning of antibodies throughout life**

***Supplementary Figures***

Samantha W.J. He ^1^, Franziska Voß ^2^, Mioara A. Nicolaie^1^, Jolanda Brummelman ^1^, Martijn D.B. van de Garde ^1^, Elske Bijvank ^1^, Martien Poelen^1^, Alienke J. Wijmenga-Monsuur^1^, Anne L. Wyllie^3^, Krzysztof Trzciński^3^, Josine Van Beek ^1^, Nynke Y. Rots ^1^, Gerco den Hartog ^1,4^, Sven Hammerschmidt ^2^# and Cécile A.C.M. van Els ^1,5^ #

^1^ Centre for Infectious Disease Control, National Institute for Public Health and the Environment, Bilthoven, The Netherlands

^2^Department of Molecular Genetics and Infection Biology, Interfaculty Institute of Genetics and Functional Genomics, Center for Functional Genomics of Microbes, University of Greifswald, Greifswald, Germany

^3^ Department of Pediatric Immunology and Infectious Diseases, Wilhelmina Children's Hospital, University Medical Center Utrecht (UMCU), Utrecht, Netherlands

^4^ Laboratory of Medical Immunology, Radboudumc, Nijmegen, The Netherlands

^5^ Infectious Diseases & Immunology, Department of Biomolecular Health Sciences, Faculty of Veterinary Medicine, Utrecht University, Utrecht, The Netherlands


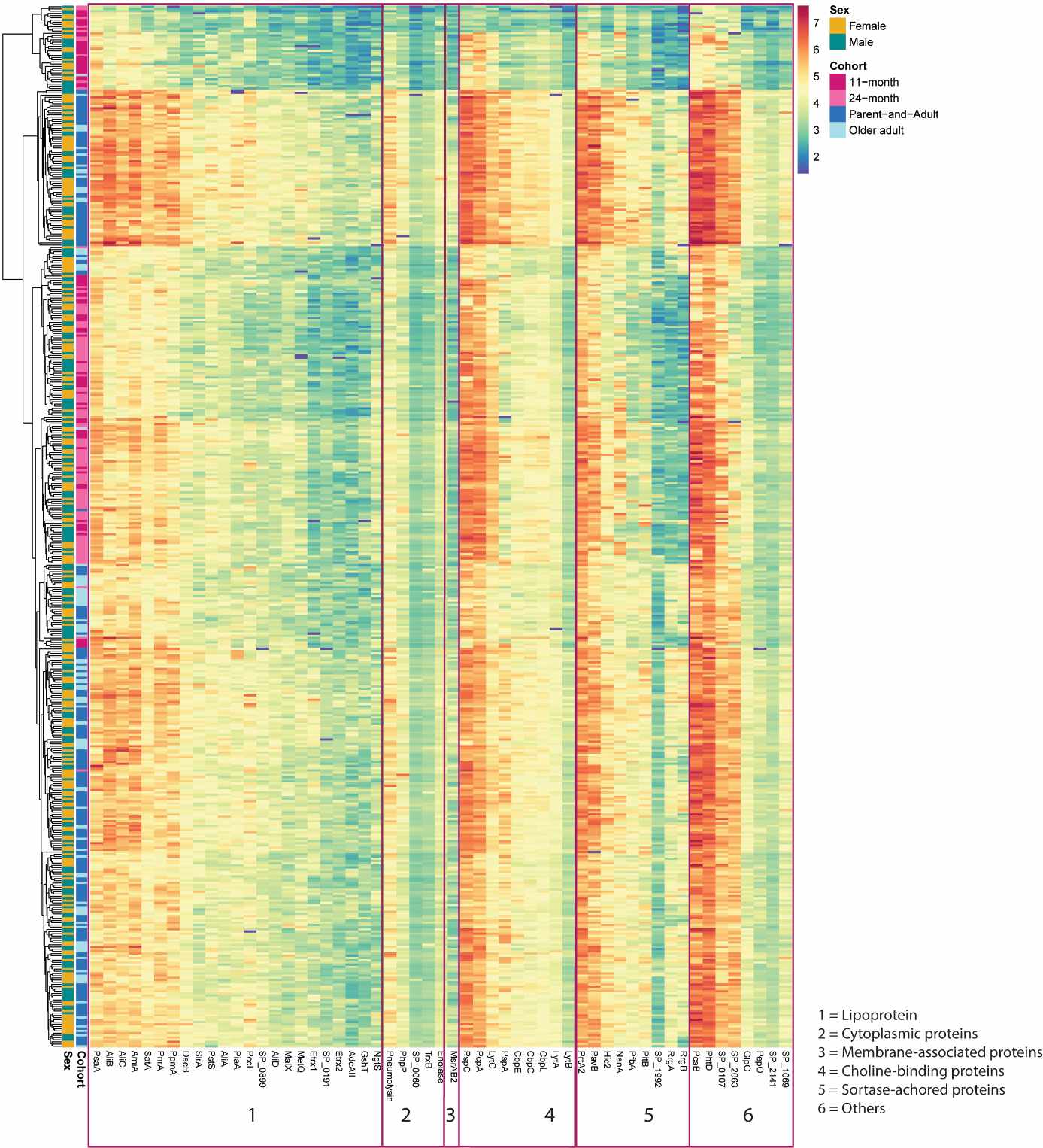


**Figure S1 Serological Profiling of Antibody Responses to Pneumococcal Proteins in age cohorts.** Heatmap of normalized and log-10 transformed IgG levels against a 55-multiplex pneumococcal protein panel in 472 serum samples. Each row represents one serum sample, and each column represents an individual protein in the panel. The colours in the heatmap range from blue to red, indicating low to high IgG levels, respectively. The age cohorts consists of 11-months old (dark pink), 24-months old (light pink), adults (consisting of parents and adults without children < 6 years old combined in dark blue), and older adults (light blue). Females and males are indicated by yellow and blue colours, respectively. Antigens are manually sorted into their respective protein classes from high to low antibody levels across all age groups.


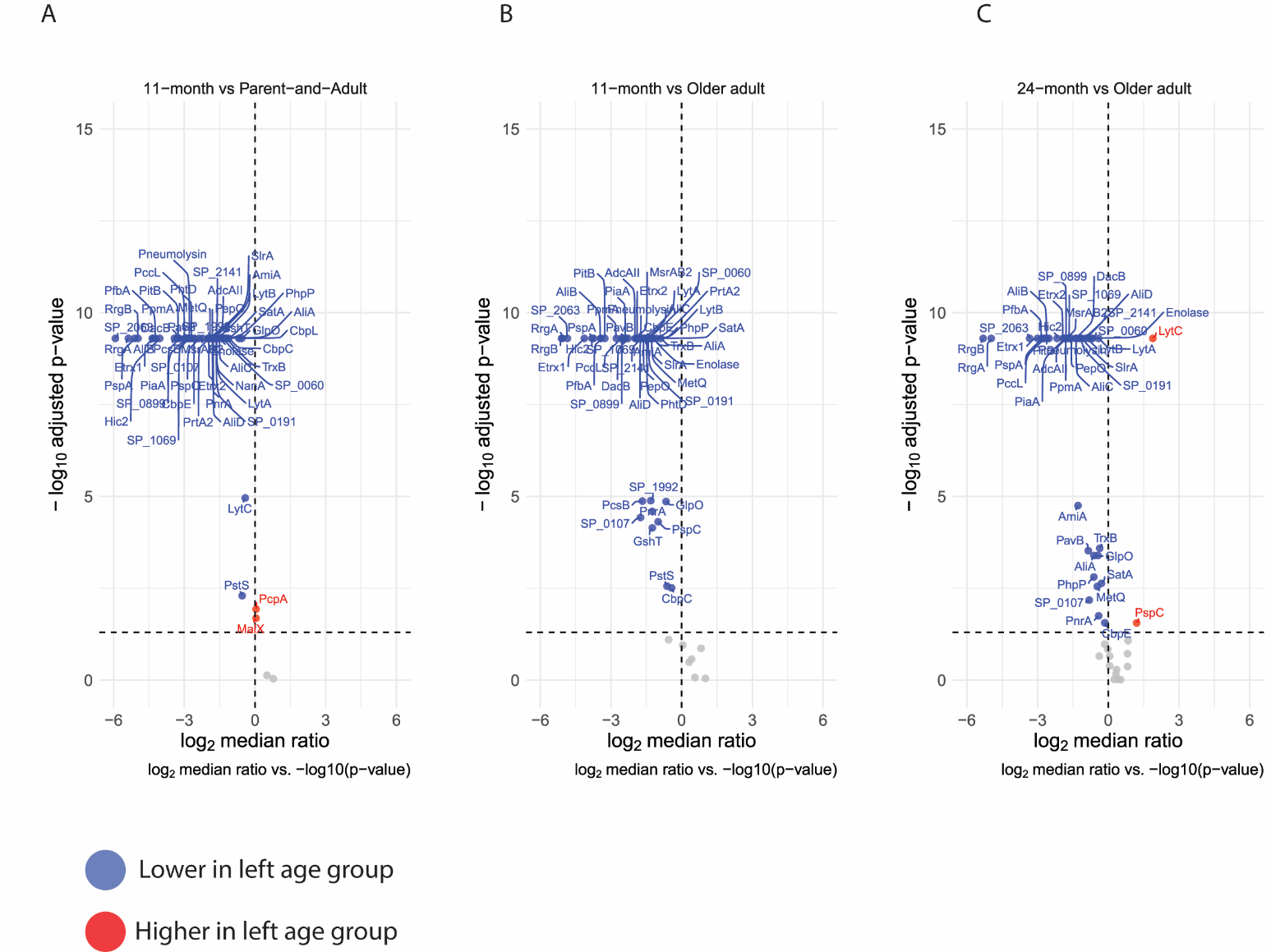


**Figure S2. Age-dependent differences in pneumococcal protein-specific IgG responses.** Volcano plots depicting log2-fold changes in IgG responses between two age groups for protein panel after controlling for confounding factors such as sex, ongoing pneumococcal carriage episodes and enhanced exposure frequencies. Y-axis depicts the log10-transformed p-value (cut-off at 0.05) on the original scale and at log10(0,05) on the transformed scale), whilst x-axis depicts the log2-fold difference in IgG levels against individual proteins between two age groups (cut-off at -1 for proteins with at least 2-fold lower IgG levels in left age group, cut-off at +1 for proteins with at least 2-fold higher IgG levels in left age group, as colour indicated). The colours of the dots indicate the log2-fold difference between the two age groups, with blue dots indicating proteins with significantly lower IgG levels in the left age group and red dots indicating proteins with significantly higher IgG levels in the left age group. Significance was based on the Wilcoxon rank sum test with Benjamin-Hochberg correction for multiple testing.


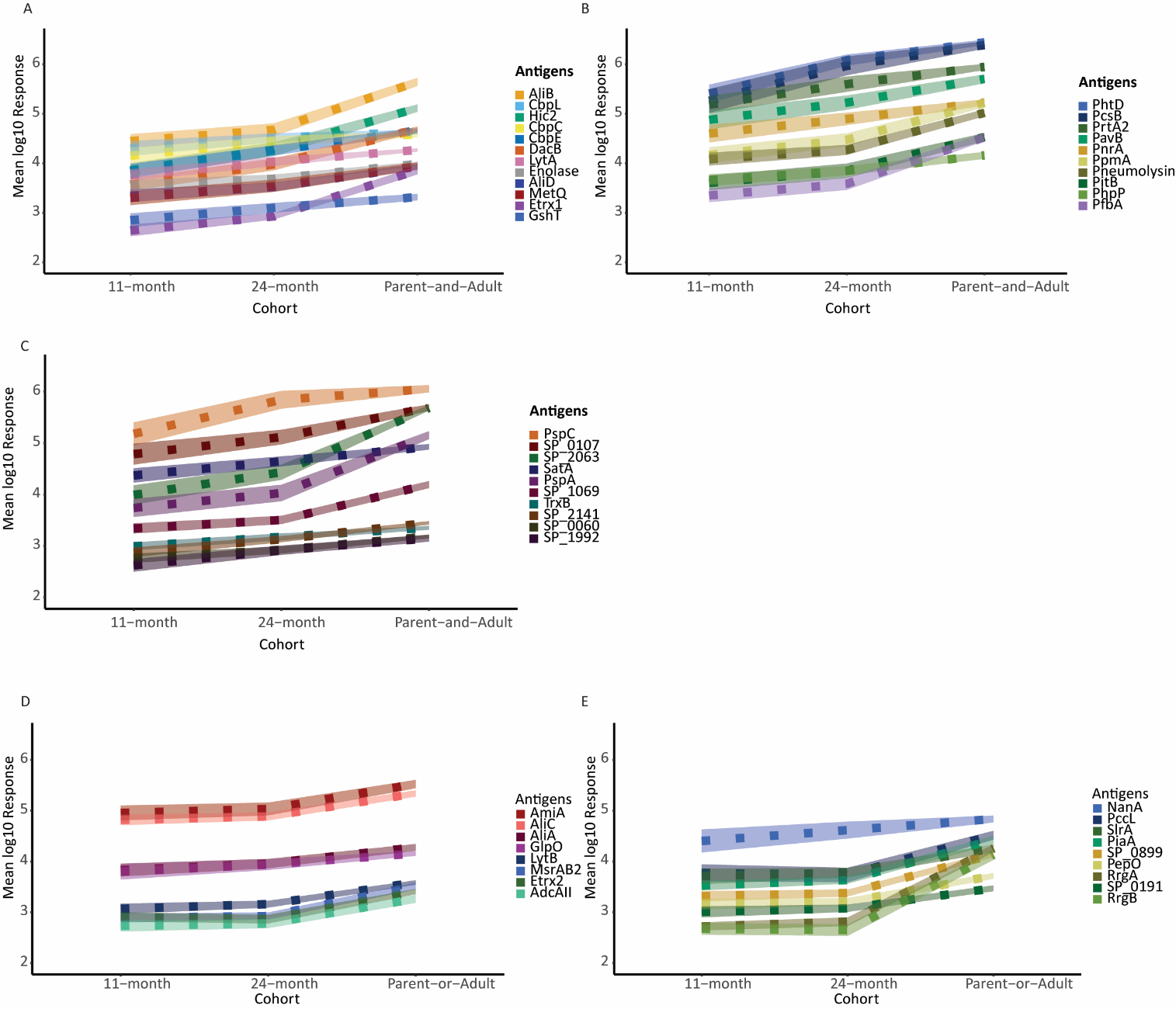


**Figure S3 Age-dependent differences in pneumococcal protein-specific IgG responses.** Line graphs depict gradual development of pneumococcal protein-specific IgG levels shown in Figure 3A, split into three graphs (A, B and C) and Figure 3C, split into two graphs (D and E), to increase visibility of the IgG levels of each antigen. Proteins depicted in legend are sorted from high (top) to low (bottom), corresponding to the depicted line in figure. Lines between cohort serve as visualization of patterns, but do not indicate longitudinal relationship.


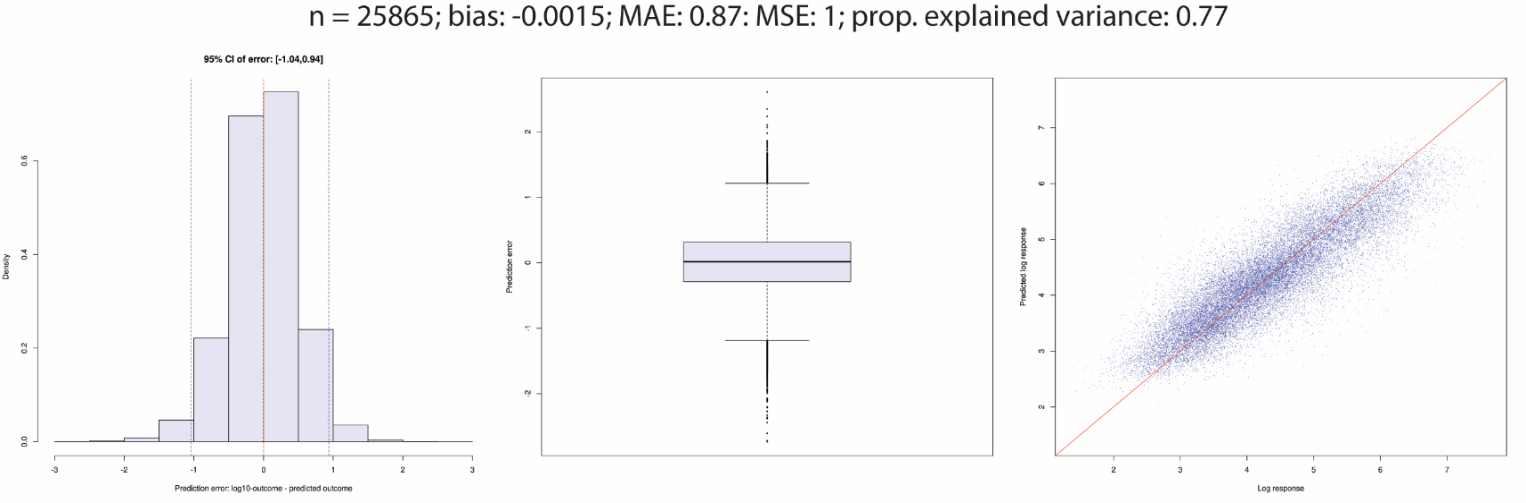


**Figure S4 Random forest analysis prediction error. (A)** The histogram and **(B)** boxplot shows the distribution of predicted response values (blue bars) generated by the random forest model. The predicted response values have a mean squared error of 1 and a mean absolute error of 0,87. **(C)** Dot plot showing the relationship between predicted and actual IgG levels. Each dot represents an individual data point, with its position along the y-axis indicating predicted IgG level and its position along the x-axis indicating actual IgG level (log response). The line of unity (y = x) is also shown for reference. The plot demonstrates the agreement between predicted and actual IgG levels, with similar levels of variability observed in both the predicted and actual data.
