# Supplementary material for: Serological Profiling of Pneumococcal Proteins Reveals Unique Patterns of Acquisition, Maintenance, and Waning of Antibodies Throughout Life
Source: J Infect Dis. 2024 Apr 29;230(6):e1299–310. doi: 10.1093/infdis/jiae216 (PMC11646596; doi:10.1093/infdis/jiae216)
Supplement: jiae216_Supplementary_Data [file jiae216_supplementary_data.zip › Table S2.docx]

**Table S2.** Overview of recombinantly expressed and purified pneumococcal protein antigens.

| No. | Protein class | Proteins (TIGR4) | New locus tag (TIGR4) | Template DNA from *S. pneumoniae* | Primer forward (5'-3') | Primer reverse (5'-3') | Restriction site | Plasmid | Tag | Purifica-tion method^a-e^ | Status | Reference |
| --- | --- | --- | --- | --- | --- | --- | --- | --- | --- | --- | --- | --- |
| 1 | Lipoprotein | AdcAII (SP_1002) | SP_RS04970 | TIGR4[1] | GGGCGCTAGCGGTCAAAAGGAAAGTCAGAC | GCGGCCAAGCTTACTTTAATTCTTCTGCTAG | *Nhe*I, *Hin*dIII | pTP1[2] | His_6_ | ^a^ |  | [3] |
| 2 |  | AliA (SP_0366) | SP_RS01790 | D39, NCTC7466 | AATTGCTAGCTCTGGATCAGGTTCAAGC | GCGCGAGCTCATTTCACATGTTTTGC | *Nhe*I, *Sac*I | pTP1[2] | His_6_ | ^a^ | Preclinical | [4,5] |
| 3 |  | AliB (SP_1527) | SP_RS07525 | D39, NCTC7466 | CGCGTGCTAGCGGAAATTCTAGCACTGCATC | GCGCGAGCTCTTATTTGACATGTTTTGCC | *Nhe*I, *Sac*I | pTP1[2] | His_6_ | ^a^ |  | [4] |
| 4 |  | AliC | - | MNZ41 | GCGCGCGCTAGCAAAAGTGAAAAGAATGC | GCGCGCGAGCTCATTTTATGTGCTTTTC | *Nhe*I, *Sac*I | pTP1[2] | His_6_ | ^a^ |  | [4] |
| 5 |  | AliD | - | MNZ41 | GCGCGCACTAGTTCAGATACAAAAACTTAC | GCGCGCAAGCTTATTTAACATGTTTTTCTGC | *Spe*I, *Hin*dIII | pTP1[2] | His_6_ | ^a^ |  | [4] |
| 6 |  | AmiA (SP_1891) | SP_RS09395 | TIGR4[1] | GCGCGCGCTAGCAGTTCTTCAAAATCATCTGATTC | GCGCGCGAGCTCTTACTTCACATGACTTGCCAATTC | *Nhe*I, *Sac*I | pTP1[2] | His_6_ | ^a^ |  | [4] |
| 7 | Lipoprotein | DacB (SP_0629) | SP_RS03085 | TIGR4[1] | GCGCGCTAGCCAAGAAAAAACAAAAAATGAAG | GGCCAAGCTTAATCGACGTAGTCTCC | *Nhe*I, *Hin*dIII | pTP1[2] | His_6_ | ^a^ | Preclinical | [6] |
| 8 |  | Etrx1 (SP_0659) | SP_RS03235 | TIGR4[1] | AAGCGCTAGCTCAGGCAAGTCCGTGACTAG | GGCCAAGCTTAGGCTAATTCCTTCAAAGTTTG | *Nhe*I, *Hin*dIII | pTP1[2] | His_6_ | ^a^ |  | [2] |
| 9 |  | Etrx2 (SP_1000) | SP_RS04960 | TIGR4[1] | AAACGCTAGCGGTGAGGAAGAAACTAAAAAG | ACGCGAGCTCCTAGTTCATTTCTTTAAATGC | *Nhe*I, *Sac*I | pTP1[2] | His_6_ | ^a^ |  | [2] |
| 10 |  | MetQ (SP_0149) | SP_RS00770 | TIGR4[1] | AAAGCATATGAGCGGCGAAAACCTGTATTTTCAGGGCGCTAGCGGAAACTCAGAAAAGAAAGC | CCAACCTTCCAAGCTTACCAAACTGGTTGATCC | *Nde*I, *Hin*dIII | pTP1[2] | His_6_ | ^a^ |  | [3] |
| 11 |  | PccL (SP_0198) | SP_RS00960 | TIGR4[1] | AAGCGCTAGCTCACAAAGAGCTCAACAGG | GGCCAAGCTTATTTAGTTAAAACGATTTGGTCCG | *Nhe*I, *Hin*dIII | pTP1[2] | His_6_ | ^a^ |  | [4] |
| 12 |  | PiaA (SP_1032) | SP_RS05120 | TIGR4[1] | GCGCGCGCTAGCTCTTCTAATTCTGTTAAAAATGAAG | GCGCGCGAGCTCTTATTTCGCATTTTTGCATGCAT | *Nhe*I, *Sac*I | pTP1[2] | His_6_ | ^a^ | Preclinical | [4,7] |
| 13 |  | PnrA (SP_0845) | SP_RS04135 | TIGR4[1] | AAGCGCTAGCGGTAACCGCTCTTCTCGTA | GGGGCCAAGCTTATTTTTCAGGAACTTTTACGC | *Nhe*I, *Hin*dIII | pTP1[2] | His_6_ | ^a^ | Preclinical | [3] |
| 14 | Lipoprotein | PpmA (SP_0981) | SP_RS04865 | TIGR4[1] | CCATGGCTAGCCACCATCACCATCACCATTCGAAAGGGTCAGAAGGTGC | TCATGGATCCGGACTATTCGTTTGATGTAC | *Nhe*I, *Bam*HI | pET11a | His_6_ | ^a^ | Preclinical | [8,9] |
| 15 |  | PsaA (SP_1650) | SP_RS08145 | TIGR4[1] | GCGCGCTAGCGGAAAAAAAGATAC | GCGCAAGCTTATTTTGCCAATCCTTCAG | *Nhe*I, *Hin*dIII | pTP1[2] | His_6_ | ^a^ | Clinical | [3] |
| 16 |  | SlrA (SP_0771) | SP_RS03765 | TIGR4[1] | TTTACTGCATATGCACCATCACCATCACCATAGCAGCGTCCAACGCAGT | CATTAGGATCCAATCGCTGGGGAAGTG | *Nde*I, *Bam*HI | pET11a | His_6_ | ^a^ |  | [8] |
| 17 |  | GshT (SP_0148) | SP_RS00765 | TIGR4[1] | GGCCACTAGTGGGGGTGCTAAGAAAGAA | GGCCAAGCTTATTTAATATCAGCTTCTGCCGG | *Spe*I, *Hin*dIII | pTP1[2] | His_6_ | ^a^ |  | [4] |
| 18 |  | SP_0191 | SP_RS00935 | TIGR4[1] | TATTTTCAGGGCGCTAGCGGACAGAAAAAAGAAACTGG | CCAACCTTCCAAGCTTATTGTTCTGTCGCGCCATTTG | *Nhe*I, *Hin*dIII | pTP1[2] | His_6_ | ^a^ |  | [3] |
| 19 |  | SP_0899 | SP_RS04440 | TIGR4[1] | GCGCGCTAGCCAACAACAACATGCTACTTC | GGCCGAGCTCTTAAAGTTTAACCCACTTATC | *Nhe*I, *Hin*dIII | pTP1[2] | His_6_ | ^a^ |  | [3] |
| 20 |  | NgtS (SP_0092) | SP_RS00475 | TIGR4[1] | ATCGGCTAGCggaaatttgacaggt | GCGCGTCGACTTAttttttgtttttcaagaattc | *Nhe*I, *Sal*I | pTP1[2] | His_6_ | ^a^ |  | This work |
| 21 | Lipoprotein | PstS (SP_2084) | SP_RS10610 | TIGR4[1] | ATTAGCTAGCggcaatcagtcagctgct | ATCGGTCGACTTAtttaatcttgtcccagg | *Nhe*I, *Sal*I | pTP1[2] | His_6_ | ^a^ |  | This work |
| 22 |  | MalX (SP_2108) | SP_RS10745 | TIGR4[1] | ATTAGCTAGCggaagcaaaactgctgat | GCGCGTCGACTTAttcaccaaatttttgtttg | *Nhe*I, *Sal*I | pTP1[2] | His_6_ | ^a^ | Preclinical | This work,  [9] |
| 23 |  | SatA (SP_1683) | SP_RS08315 | TIGR4[1] | TATAGCTAGCggcaattctggcggaagt | GCGCGTCGACTTAttgtttcatagctttttt | *Nhe*I, *Sal*I | pTP1[2] | His_6_ | ^a^ | Preclinical | This work |
| 24 | Choline-binding protein | CbpC (SP_0377) | SP_RS01845 | TIGR4[1] | GCGCGCGCTAGCAATACCACAGGTGGCCGATTTG | GCGCGCAAGCTTAAATCCACTCACCAGATGAGGCG | *Nhe*I, *Hin*dIII | pTP1[2] | His_6_ | ^b^ |  | [4] |
| 25 |  | CbpE (SP_0930) | SP_RS04600 | TIGR4[1] | CCGAATTCAAGGAGATTAACATATGCAAGAAAGTTCAGGAAATAAAATCC | CGGGATCCTCATTATGTAGTTTTAATTGTAGCAGATTTCTC | *Nde*I, *Bam*HI | pT7-7 | His_6_ | ^b^ |  | [10] |
| 26 |  | CbpL (SP_0667) | SP_RS03275 | TIGR4[1] | GCGCGCTAGCGAAGAAAACATCCATTTTTC | GGCCGAGCTCTTAATCATCTAAATGATCAATGG | *Nhe*I, *Sac*I | pTP1[2] | His_6_ | ^c^ | Preclinical | [11,12] |
| 27 |  | LytA (SP_1937) | SP_RS09740 | D39, NCTC7466 | GCGCGCTAGCGAAATTAATGTGAGTAAATTAAGAACAG | GCGCAAGCTTATTTTACTGTAATCAAGCCATCTGGC | *Nhe*I, *Hin*dIII | pTP1[2] | His_6_ | ^b^ | Preclinical | [4] |
| 28 | Choline-binding protein | LytB (SP_0965) | SP_RS04785 | TIGR4[1] | GCGCGCGCTAGCGGCCTGCATTTTGAT | GCGCGCGAGCTCTTAATCTTTGCCACCTAGCTTCTC | *Nhe*I, *Sac*I | pTP1[2] | His_6_ | ^b^ |  | [4] |
| 29 |  | LytC (SP_0377) | SP_RS07755 | TIGR4[1] | GCGCGCGCTAGCGCAAATGAAACTGAAGTAGC | GCGCGCAAGCTTAATACCAAACGCTGACATCTAC | *Nhe*I, *Hin*dIII | pTP1[2] | His_6_ | ^b^ | Preclinical | [4] |
| 30 |  | PcpA (SP_2136) | SP_RS10900 | TIGR4[1] | GGCCGCTAGCCCTAGTTCGGAAGTAATC | GCGCGCGAGCTCTTATCCTACCCACTCACCGTTAG | *Nhe*I, *Sac*I | pTP1[2] | His_6_ | ^b^ | Clinical | [4,13] |
| 31 |  | PspA (SP_0117)  QP2 | SP_RS00595 | D39, NCTC7466, serotype 2 | GGATCCGAAGAAGAATCTCCCGTAGCC | AAGCTTATTAACTGCTTTCTTAAGGTC | *Bam*HI, *Hin*dIII | pQE30 | His_6_ | ^a^ | Clinical | [14] |
| 32 |  | PspC (SP_2190)  SH2 | SP_RS11185 | NCTC 7465, serotype 1 | GCGCGCGCGCGCGGATCCACAGAGAACGAGGGAAGTACCC | AAGCTTTTCTTTAACTTTATCTTCTTCTGCTG | *Bam*HI, *Hin*dIII | pQE30 | His_6_ | ^a^ | Prelinical | [15] |
| 33 | Sortase-anchored protein | Hic 2  (PspC-like) | - | A66, NCTC7978, serotype 3 | GCGCGCTAGCACAGAGAAGGAGGTAACTACCC | GCGCAAGCTTATTTAGTGGAGGAGCCTGAATTCG | *Nhe*I, *Hin*dIII | pTP1[2] | His_6_ | ^a^ |  | [16] |
| 34 |  | NanA (SP_1326) | SP_RS08365 | TIGR4[1] | GCGCGCGCTAGCCAGGAGACTGAAACTTCTGAAG | GCGCGCAAGCTTAGATCTTCAATCTTAAATG | *Nhe*I, *Hin*dIII | pTP1[2] | His_6_ | ^a^ | Preclinical | [4] |
| 35 | Sortase-anchored protein | PavB (SP_0082) SSURE 2+3 | SP_RS00425 | TIGR4[1] | GGATCCAAAGACAGTATCGATGTTCCAGC | CTGCAGGTTTATGTTAATAGTGACTTTTTTAG- | *Bam*HI, *Pst*I | pQE30 | His_6_ | ^a^ |  | [17] |
| 36 |  | PfbA (SP_1833) | SP_RS09095 | TIGR4[1] | GCGCGCGCTAGCGATGAAGTTGTTACTAGTTCTTC | GCGCAAGCTTATTTTTGTTTTACATCTAC | *Nhe*I, *Hin*dIII | pTP1[2] | His_6_ | ^a^ |  | [4] |
| 37 |  | PitB (spt_1059) | - | Taiwan19F-14, serotype 19F | GCGCGCGGATCCGATAATTCAGCAATAACCAAAG | GCGCGCCTGCAGGTCGTCGATTTTGTTAGTAAC | *Bam*HI, *Pst*I | pQE30 | His_6_ | ^a^ |  | [4] |
| 38 |  | PrtA2 (SP_0641) | SP_RS03145 | TIGR4[1] | CCAGGCTAGCTCACCTAGACAACAGGGAGCA | GAGTGAGCTCTCAGTGGTTCCAATTCCCAGCAA | *Nhe*I, *Sac*I | pTP1[2] | His_6_ | ^a^ |  | [4] |
| 39 |  | RrgA (SP_0462) | SP_RS02280 | TIGR4[1] | GCGCGCTAGCGAAACGCCTGAAACCAGT | GCGCGAGCTCTTATTCTCTCTTTGGAGGAATAG | *Nhe*I, *Sac*I | pTP1[2] | His_6_ | ^d^ | Preclinical | [4,18,19] |
| 40 |  | RrgB (SP_0463) | SP_RS02285 | TIGR4[1] | GCGCGCGGATCCGAAACGCCTGAAACCAGTC | GCGCGCGCATGCTTCTCTCTTTGGAGGAATAGG | *Bam*HI, *Sph*I | pQE30 | His_6_ | ^a^ | Preclinical | [4,18,19] |
| 41 |  | SP_1992 | SP_RS10010 | TIGR4[1] | GGCCGCTAGCACGATTCTAGGAAAAGATACAG | GGCCGAGCTCTAATTGTTTGCCAGCAGG | *Nhe*I, *Sac*I | pTP1[2] | His_6_ | ^a^ |  | [4] |
| 42 | Cytoplasmic protein | PhpP (SP_1733) | SP_RS08575 | TIGR4[1] | GGCCGCTAGCGAAATTTCATTATTAACAG | GCGCGCGAGCTCTTATTCTGCATCCTCCTCGTTCATAG | *Nhe*I, *Sac*I | pTP1[2] | His_6_ | ^a^ |  | [4] |
| 43 |  | Pneumolysin (SP_1923) | SP_RS09670 | TIGR4[1] | CGGGATCCGCAAATAAAGCAGTAAATGAC | GCGGTACCCTAGTCATTTTCTACCTGAG | *Bam*HI, *Kpn*I | pASK-IBA5 | Strep | ^e^ | Clinical | [4] |
| 44 |  | TrxB (SP_1458) | SP_RS07165 | TIGR4[1] | GCGCGCGCTAGCTACGATACTATTATTATCGGTG | GCGCGCAAGCTTAACTATGTTCTGTAATGA | *Nhe*I, *Hin*dIII | pTP1[2] | His_6_ | ^a^ |  | [4] |
| 45 |  | SP_0060 | SP_RS00335 | TIGR4[1] | TAGCGCTAGCacacgatttgagatacga | TAGCGTCGACTTAtaagttttccccctttat | *Nhe*I, *Sal*I | pTP1[2] | His_6_ | ^a^ |  | This work |
| 46 |  | Enolase (SP_1128) | SP_RS05590 | TIGR4[1] | GGATCCTTGTCAATTATTACTGATGTTTACGC | AAGCTTTTATTTTTTAAGGTTGTAGAATGATTTC | *Bam*HI, *Hin*dIII | pQE30 | His_6_ | ^a^ |  | [20] |
| 47 | Membrane protein | MsrAB2 (SP_0660) | SP_RS03240 | TIGR4[1] | ATATGCTAGCGGGCAGACAGATGCCTCG | CCCGGGCCGAGCTCTTAATCAACATAATCTAG | *Nhe*I, *Sac*I | pTP1[2] | His_6_ | ^a^ |  | [2] |
| 48 | Others | PcsB (SP_2216) | SP_RS11315 | RH1 | GAAACGACTGATGACAAAATTG | GATCGAATTCTTAATCTGCATAAATATATGTAAC | *Eco*RI | pRSET A | CHiC* | ^a^ | Clinical | [21] |
| 49 | Others | PhtD (SP_1003) | SP_RS04975 | TIGR4[1] | GCGCGCACTAGTGGTCAGGTTAAGAAAGAGTCTAATC | GCGCGCAAGCTTACTGTATAGGAGCCGGTTGAC | *Spe*I, *Hin*dIII | pTP1[2] | His_6_ | ^d^ | Clinical | [4] |
| 50 |  | SP_1069 | SP_RS05285 | TIGR4[1] | GCGCGCGCTAGCCAGAATAATAAGGATGAGAAGAAAATAAC | GCGCGCAAGCTTATTCGATGACTTGTCCTGCTTC | *Nhe*I, *Hin*dIII | pTP1[2] | His_6_ | ^a^ |  | [4] |
| 51 |  | PepO (SP_1647) | SP_RS08130 | TIGR4[1] | GCGCGCACTAGTACACGTTATCAAGATGA | GCGCGCAAGCTTACCAAATAATCACGCGCTCCTC | *Spe*I, *Hin*dIII | pTP1[2] | His_6_ | ^a^ |  | [4] |
| 52 |  | SP_0107 | SP_RS00545 | TIGR4[1] | GCGCGCGCTAGCCAAGAATCATCAACTTAC | GCGCGCAAGCTTAATACCAGCCATTGTTAAGCCA | *Nhe*I, *Hin*dIII | pTP1[2] | His_6_ | ^a^ |  | [4] |
| 53 |  | SP_2063 | SP_RS10430 | TIGR4[1] | GCGCGCGCTAGCGAAGAAGTTCTTTGGACTGCAC | GCGCGCAAGCTTATCCATTCATTGAAACGTGAAC | *Nhe*I, *Hin*dIII | pTP1[2] | His_6_ | ^a^ |  | [4] |
| 54 |  | SP_2141 | SP_RS10925 | TIGR4[1] | ATTAGCTAGCgctgtcactcagtctg | ATTAGTCGACTTAtgtcgcaatggtatgc | *Nhe*I, *Sal*I | pTP1[2] | His_6_ | ^a^ |  | This work |
| 55 |  | GlpO (SP_2185) | SP_RS11160 | TIGR4[1] | ATTAGCTAGCagcggtcttgagactg | GCGCGTCGACTTAattttttaattctgctaa | *Nhe*I, *Sal*I | pTP1[2] | His_6_ | ^a^ | Preclinical | This work |

The purification methods are indicated by the following superscript letters as follows: ^a^, HisTrap Ni-NTA HP 1-ml column with ÄKTApurifier liquid chromatography system (GE Healthcare GmbH); ^b^, DEAE-cellulose column; ^c^, DEAE-cellulose column plus HisTrap Ni-NTA HP 1-ml column with ÄKTApurifier liquid chromatography system (GE Healthcare GmbH); ^d^, HisTrap Ni-NTA HP 1-ml column with ÄKTApurifier liquid chromatography system (GE Healthcare GmbH) plus gel filtration Superdex 200; ^e^, StrepTactin Sepharose High Performance (GE Healthcare GmbH). The asterisk in the Tag column indicates CHiC, a choline-binding histidine combination tag.

**References:**

1. Tettelin, H.; Nelson, K.E.; Paulsen, I.T.; Eisen, J.A.; Read, T.D.; Peterson, S.; Heidelberg, J.; DeBoy, R.T.; Haft, D.H.; Dodson, R.J.; et al. Complete genome sequence of a virulent isolate of *Streptococcus pneumoniae*. *Science* **2001**, *293*, 498-506, doi:10.1126/science.1061217.

2. Saleh, M.; Bartual, S.G.; Abdullah, M.R.; Jensch, I.; Asmat, T.M.; Petruschka, L.; Pribyl, T.; Gellert, M.; Lillig, C.H.; Antelmann, H.; et al. Molecular architecture of Streptococcus pneumoniae surface thioredoxin-fold lipoproteins crucial for extracellular oxidative stress resistance and maintenance of virulence. *EMBO Mol Med* **2013**, *5*, 1852-1870, doi:10.1002/emmm.201202435.

3. Voss, F.; Kohler, T.P.; Meyer, T.; Abdullah, M.R.; van Opzeeland, F.J.; Saleh, M.; Michalik, S.; van Selm, S.; Schmidt, F.; de Jonge, M.I.; et al. Intranasal Vaccination With Lipoproteins Confers Protection Against Pneumococcal Colonisation. *Front Immunol* **2018**, *9*, 2405, doi:10.3389/fimmu.2018.02405.

4. Seinen, J.; Engelke, R.; Abdullah, M.R.; Voss, F.; Michalik, S.; Dhople, V.M.; Dieperink, W.; de Smet, A.; Volker, U.; van Dijl, J.M.; et al. Sputum Proteome Signatures of Mechanically Ventilated Intensive Care Unit Patients Distinguish Samples with or without Anti-pneumococcal Activity. *mSystems* **2021**, *6*, doi:10.1128/mSystems.00702-20.

5. van Beek, L.F.; Surmann, K.; van den Berg van Saparoea, H.B.; Houben, D.; Jong, W.S.P.; Hentschker, C.; Ederveen, T.H.A.; Mitsi, E.; Ferreira, D.M.; van Opzeeland, F.; et al. Exploring metal availability in the natural niche of Streptococcus pneumoniae to discover potential vaccine antigens. *Virulence* **2020**, *11*, 1310-1328, doi:10.1080/21505594.2020.1825908.

6. Abdullah, M.R.; Gutierrez-Fernandez, J.; Pribyl, T.; Gisch, N.; Saleh, M.; Rohde, M.; Petruschka, L.; Burchhardt, G.; Schwudke, D.; Hermoso, J.A.; et al. Structure of the pneumococcal l,d-carboxypeptidase DacB and pathophysiological effects of disabled cell wall hydrolases DacA and DacB. *Mol Microbiol* **2014**, *93*, 1183-1206, doi:10.1111/mmi.12729.

7. Jomaa, M.; Terry, S.; Hale, C.; Jones, C.; Dougan, G.; Brown, J. Immunization with the iron uptake ABC transporter proteins PiaA and PiuA prevents respiratory infection with Streptococcus pneumoniae. *Vaccine* **2006**, *24*, 5133-5139, doi:10.1016/j.vaccine.2006.04.012.

8. Hermans, P.W.; Adrian, P.V.; Albert, C.; Estevao, S.; Hoogenboezem, T.; Luijendijk, I.H.; Kamphausen, T.; Hammerschmidt, S. The streptococcal lipoprotein rotamase A (SlrA) is a functional peptidyl-prolyl isomerase involved in pneumococcal colonization. *J Biol Chem* **2006**, *281*, 968-976, doi:10.1074/jbc.M510014200.

9. Narciso, A.R.; Iovino, F.; Thorsdottir, S.; Mellroth, P.; Codemo, M.; Spoerry, C.; Righetti, F.; Muschiol, S.; Normark, S.; Nannapaneni, P.; et al. Membrane particles evoke a serotype-independent cross-protection against pneumococcal infection that is dependent on the conserved lipoproteins MalX and PrsA. *Proc Natl Acad Sci U S A* **2022**, *119*, e2122386119, doi:10.1073/pnas.2122386119.

10. Lagartera, L.; Gonzalez, A.; Stelter, M.; Garcia, P.; Kahn, R.; Menendez, M.; Hermoso, J.A. Crystallization and preliminary X-ray diffraction studies of the pneumococcal teichoic acid phosphorylcholine esterase Pce. *Acta Crystallogr Sect F Struct Biol Cryst Commun* **2005**, *61*, 221-224, doi:10.1107/S1744309105001636.

11. Gutierrez-Fernandez, J.; Saleh, M.; Alcorlo, M.; Gomez-Mejia, A.; Pantoja-Uceda, D.; Trevino, M.A.; Voss, F.; Abdullah, M.R.; Galan-Bartual, S.; Seinen, J.; et al. Modular Architecture and Unique Teichoic Acid Recognition Features of Choline-Binding Protein L (CbpL) Contributing to Pneumococcal Pathogenesis. *Sci Rep* **2016**, *6*, 38094, doi:10.1038/srep38094.

12. Kazemian, H.; Afshar, D.; Garcia, E.; Pourmand, M.R.; Jeddi-Tehrani, M.; Aminharati, F.; Shokri, F.; Yazdi, M.H. CbpM and CbpG of Streptococcus Pneumoniae Elicit a High Protection in Mice Challenged with a Serotype 19F Pneumococcus. *Iran J Allergy Asthma Immunol* **2018**, *17*, 574-585.

13. Bologa, M.; Kamtchoua, T.; Hopfer, R.; Sheng, X.; Hicks, B.; Bixler, G.; Hou, V.; Pehlic, V.; Yuan, T.; Gurunathan, S. Safety and immunogenicity of pneumococcal protein vaccine candidates: monovalent choline-binding protein A (PcpA) vaccine and bivalent PcpA-pneumococcal histidine triad protein D vaccine. *Vaccine* **2012**, *30*, 7461-7468, doi:10.1016/j.vaccine.2012.10.076.

14. Hammerschmidt, S.; Bethe, G.; Remane, P.H.; Chhatwal, G.S. Identification of pneumococcal surface protein A as a lactoferrin-binding protein of *Streptococcus pneumoniae*. *Infect Immun* **1999**, *67*, 1683-1687.

15. Hammerschmidt, S.; Tillig, M.P.; Wolff, S.; Vaerman, J.P.; Chhatwal, G.S. Species-specific binding of human secretory component to SpsA protein of *Streptococcus pneumoniae* via a hexapeptide motif. *Mol Microbiol* **2000**, *36*, 726-736, doi:10.1046/j.1365-2958.2000.01897.x.

16. Kohler, S.; Hallstrom, T.; Singh, B.; Riesbeck, K.; Sparta, G.; Zipfel, P.F.; Hammerschmidt, S. Binding of vitronectin and Factor H to Hic contributes to immune evasion of *Streptococcus pneumoniae* serotype 3. *Thromb Haemost* **2015**, *113*, 125-142, doi:10.1160/TH14-06-0561.

17. Jensch, I.; Gamez, G.; Rothe, M.; Ebert, S.; Fulde, M.; Somplatzki, D.; Bergmann, S.; Petruschka, L.; Rohde, M.; Nau, R.; et al. PavB is a surface-exposed adhesin of *Streptococcus pneumoniae* contributing to nasopharyngeal colonization and airways infections. *Mol Microbiol* **2010**, *77*, 22-43, doi:10.1111/j.1365-2958.2010.07189.x.

18. Harfouche, C.; Filippini, S.; Gianfaldoni, C.; Ruggiero, P.; Moschioni, M.; Maccari, S.; Pancotto, L.; Arcidiacono, L.; Galletti, B.; Censini, S.; et al. RrgB321, a fusion protein of the three variants of the pneumococcal pilus backbone RrgB, is protective in vivo and elicits opsonic antibodies. *Infect Immun* **2012**, *80*, 451-460, doi:10.1128/IAI.05780-11.

19. Moschioni, M.; De Angelis, G.; Harfouche, C.; Bizzarri, E.; Filippini, S.; Mori, E.; Mancuso, G.; Doro, F.; Barocchi, M.A.; Ruggiero, P.; et al. Immunization with the RrgB321 fusion protein protects mice against both high and low pilus-expressing Streptococcus pneumoniae populations. *Vaccine* **2012**, *30*, 1349-1356, doi:10.1016/j.vaccine.2011.12.080.

20. Bergmann, S.; Rohde, M.; Chhatwal, G.S.; Hammerschmidt, S. alpha-Enolase of *Streptococcus pneumoniae* is a plasmin(ogen)-binding protein displayed on the bacterial cell surface. *Mol Microbiol* **2001**, *40*, 1273-1287, doi:10.1046/j.1365-2958.2001.02448.x.

21. Stamsas, G.A.; Havarstein, L.S.; Straume, D. CHiC, a new tandem affinity tag for the protein purification toolbox. *J Microbiol Methods* **2013**, *92*, 59-63, doi:10.1016/j.mimet.2012.11.003.
